# OA05 Clinical presentation, risk factors and prognosis of MDA5-positive JDM; clinical diversity and red flags

**DOI:** 10.1093/rap/rkac066.005

**Published:** 2022-09-28

**Authors:** Kirsty McLellan, Muthana Al-Obaidi, Sandrine Compeyrot-Lacassagne, Clarissa Pilkington, Elena Moraitis, Charalampia Papadopoulou

**Affiliations:** Great Ormond Street Hospital, London, United Kingdom; Great Ormond Street Hospital, London, United Kingdom; Great Ormond Street Hospital, London, United Kingdom; Great Ormond Street Hospital, London, United Kingdom; Great Ormond Street Hospital, London, United Kingdom; Great Ormond Street Hospital, London, United Kingdom

## Abstract

**Introduction/Background:**

MDA5-positive juvenile dermatomyositis (JDM) represents a distinct clinical phenotype associated with skin and oral ulceration, milder muscle involvement and a higher incidence of interstitial lung disease (ILD) and severe rapidly progressive ILD (RP-ILD).

**Description/Method:**

The objectives were to describe the presenting clinical characteristics of patients with MDA5-positive JDM, the incidence of ILD and risk factors associated with development of RP-ILD. Retrospective clinical notes review of patients with MDA5-positive JDM managed at Great Ormond Street Hospital (GOSH) over a 24-year period.

**Discussion/Results Demographics:**

Twenty-five patients were managed at our centre with MDA5-positive JDM. One additional patient was excluded as no notes available. Sixty-four % females. Sixty % were White British, 20% Black African, 8% Mixed, 8% Asian, 8% European and 4% South Asian. Median age of presentation was 10.0 years (IQR 6.9,12.3). Diagnostic delay was significant, with median 14 weeks (8,26) between initial symptoms and diagnosis. 24% of this cohort were also Ro-52 positive.

**Presentation:** All patients had skin involvement; 52% ulcerating skin disease, 64% heliotrope rash and 100% Gottrons papules on initial presentation. Forty-four % had peri-ungal changes, 35% digital pitting/infarcts and 64% nailfold changes. 40% had peri-orbital oedema. Muscle involvement was present in all, with median CMAS 40/52 (36,44) and MMT8 57/80 (51,62). Eighty-eight % had arthritis; initial active joint count was available for 16 patients with median 5.5 joints (3,16). 40% had gastrointestinal symptoms; fever, weight loss and mouth ulcers were present in 52%, 52% and 60% respectively. Twenty-four % had respiratory symptoms at diagnosis.

**Interstitial lung disease:** 52% were diagnosed with ILD and a further 20% had abnormal pulmonary CT scans not thought to be diagnostic of ILD. 13% (2 patients) had RP-ILD; both of whom died despite immunosuppression and extracorporeal membrane oxygenation. There were no other deaths amongst this cohort. No respiratory involvement was identified in 28%. Of 19 patients who had a CT chest at diagnosis, 84% were abnormal. Only 1 patient developed respiratory involvement during disease course whilst on treatment; the other 93% with ILD had evidence of ILD at diagnosis. Out of 58 putative variables, 4 risk factors were associated with development of RP-ILD which reached statistical significance, a further 3 factors were significantly protective, as is shown in Table 1. 2 patients had pneumocystis pneumonia (PCP), both of whom required intensive care and one associated with death.

**Key learning points/Conclusion:**

Skin and muscle involvement were identified in all patients, with the majority also presenting with ulcerating skin disease, oral ulceration and arthritis. The majority of this cohort had lung involvement and 2 patients died of RP-ILD. Four risk factors were found to be predictive of RP-ILD and a further 3 protective factors were identified. Rapid deterioration of respiratory symptoms was associated with PCP, while ILD was unlikely to develop whilst on treatment if not present at diagnosis. These findings need to be validated in a larger cohort of patients.

**OA05 Table 1:** shows risk factors at presentation which were found to be significantly associated with RP-ILD

	Factor	p value
Risk factor	Periungal erythema	0.024
	Heliotrope rash	0.06
	Respiratory symptoms	0.017
	Haemoglobin	0.0129
Protective factor	Normal CT	0.01
	Normal lung function	0.008
	Normal CXR	0.005

